# Editorial: Biomarkers and early warning scores: the time for high-precision emergency medicine

**DOI:** 10.3389/fpubh.2023.1349881

**Published:** 2024-01-08

**Authors:** Ancor Sanz-García, Raúl López-Izquierdo, Francisco Martín-Rodríguez

**Affiliations:** ^1^Faculty of Health Sciences, Universidad de Castilla la Mancha, Talavera de la Reina, Spain; ^2^Department of Emergency, Hospital Universitario Rio Hortega, Valladolid, Spain; ^3^CIBER de Enfermedades Respiratorias, Instituto de Salud Carlos III, Madrid, Spain; ^4^Faculty of Medicine, Universidad de Valladolid, Valladolid, Spain; ^5^Advanced Life Support, Emergency Medical Services (SACYL), Valladolid, Spain

**Keywords:** emergency medicine, biomarkers, early warning score, prehospital, emergency department

The research field of emergency medicine is a broader area that generally entails several diseases. In addition, the complexity is even greater considering that clinical practice can be performed inside the hospital, by emergency departments (ED), or out-of-hospital, by emergency medical services (EMS). A critical factor commonly faced by the whole range of professionals that constitute the emergency medicine field is the early identification of patients at risk of clinical deterioration. Briefly, those professionals must confront time-dependent decisions under limited information, and sometimes with limited resources, with life-threatening conditions.

Actually, there are biomarker and early warning scores (EWS), designed to provide more information regarding the patient status. Both have shown their utility to determine the clinical impairment of patients. The aim of this Research Topic was to shed light on the biomarker and EWS used in emergency medicine, and includes brief research reports, original research, reviews, and systematics reviews.

Due to the huge number of conditions faced in emergency field, the different works presented in this Research Topic deal with the prediction of a wide range of diseases: infection Risk prediction by using machine learning-based techniques (Feng T. et al.), COVID-19 (Fu et al.; Xiao et al.; Nogueira et al.; Wang et al.; Roy-Vallejo et al.), poisoning (Yu et al.), trauma alone (Li et al.) or trauma complicated with sepsis (Feng K. et al.), acute aortic dissection (Chen et al.), cerebrovascular diseases (Deguchi et al.), and neurological patients (Donoso-Calero et al.). There were also studies describing biomarkers not for particular diseases, but for all patients admitted to the ICU (Tang et al.). In the collection presented here, other elements have also been studied, such as the assessment of overcrowding in emergency departments, which also influences the quality of care, the weekday or season showed to be important for the ED workload (Hitzek et al.). In this sense, previous triage by phone (Katayama et al.) could help to improve the always oversaturated ED. Or even, the proposal of one of the studies, in which the authors describe the utility of using a syndromic surveillance after a catastrophic event (Fernandez et al.).

As the different studies in this Research Topic shown, there are several EWS. Therefore, a key question arises: which of them is the most valuable? This was answered, at least for the prehospital setting, by one of the studies. The authors presented a systematic review that concludes that National Early warning Score (NEWS) is the most suitable for out-of-hospital (Burgos-Esteban et al.). Another work performed a critical review of the different predicting models that exist in the context of COVID-19 pandemic (Botz et al.).

To conclude, both EWS and biomarkers are a reality in the field of emergency medicine. They are tools under continuous development and research. However, many of them are already fully integrated in decision making, which due to its complexity, must take into account all the available evidence. Finally, the variety of topics covered in this collection demonstrates the great complexity and difficulty involved in this health specialty.

## Author contributions

AS-G: Writing – original draft, Writing – review & editing. RL-I: Writing – original draft, Writing – review & editing. FM-R: Writing – original draft, Writing – review & editing.

